# Amantadine reduces sepsis-induced brain injury via NLRP3/caspase-1 inflammasome activation

**DOI:** 10.22038/ijbms.2025.81436.17626

**Published:** 2025

**Authors:** Pınar Karabacak, Ahmet Bindal, Mustafa Soner Ozcan, Ilter Ilhan, Muhammet Yusuf Tepebasi, Melih Arlioğlu, Rumeysa Taner, Halil Asci

**Affiliations:** 1 Department of Anesthesiology and Reanimation, Suleyman Demirel University, Isparta, Türkiye; 2 Department of Medical Biochemistry, Faculty of Medicine, Suleyman Demirel University, Isparta, Türkiye; 3 Department of Medical Genetics, University of Suleyman Demirel, Isparta, Türkiye; 4 Department of Pharmacology, Faculty of Medicine, Suleyman Demirel University, Isparta, Türkiye; 5 Department of Bioengineering, Institute of Science and Technology, Suleyman Demirel University Isparta, Türkiye

**Keywords:** Amantadine, Apoptosis, Sepsis, Sepsis-associated – encephalopathy, Systemic inflammation

## Abstract

**Objective(s):**

Sepsis, a severe consequence of infection leading to organ failure, incites damage in frequently affected brain tissue through inflammation and oxidative stress. This study aimed to assess the effectiveness of amantadine, an N-methyl-D-aspartate (NMDA) receptor antagonist, in mitigating sepsis-induced brain damage.

**Materials and Methods:**

Thirty-two Wistar albino male rats were allocated into four groups: control, LPS (lipopolysaccharide 5 mg/kg, intraperitoneal, single-dose), LPS + amantadine, and amantadine alone. Six hours post-LPS administration, rats were euthanized under anesthesia. The neutrophilic infiltration and necrosis reaction were assessed in lung tissues through histopathological analysis, while expressions of interferon-alpha (IFN-α), caspase-3 (Cas-3), and Tumor necrosis factor-alpha (TNF-α) were examined using the immunohistochemical method. Levels of biochemical total anti-oxidant status (TAS), total oxidant status (TOS), and oxidative stress index (OSI) were evaluated via the ELISA method. IL-1β, Cas-1, NLRP3, and IL-18 were evaluated via real-time qPCR.

**Results:**

The LPS group exhibited histopathologically significant hyperemia, increased septal tissue thickness, hemorrhage, and inflammatory cell infiltrates, and increased IFN-α, Cas-3, TNF-α immunohistochemical expressions, and IL-1 beta, IL-18, NLRP3, and Cas-1, gene expressions compared to the control group. All these findings were significantly reversed with amantadine treatment.

**Conclusion:**

The pathophysiology of brain damage due to systemic inflammation is complex. Our findings suggest that amantadine reduces neuronal injury in the brain by alleviating oxidative stress and inflammation. Notably, amantadine’s efficacy appears to extend beyond NMDA receptors, implicating involvement in alternative pathways, such as Cas-1 activation by the NLRP3 inflammasome.

## Introduction

Sepsis, a life-threatening condition affecting nearly 20 million patients globally each year, arises from a dysregulated immune response to infection, ultimately leading to organ failure. The high mortality rate associated with sepsis underscores the urgent need for novel therapeutic strategies, given the limitations of current treatment approaches for this complex hyperinflammatory syndrome and its related complications (1-3). 

Sepsis-induced organ failure manifests in a variety of complications, including encephalopathy, kidney failure, liver failure, and cardiomyopathy. Sepsis-associated encephalopathy (SAE) is particularly concerning, with an incidence reported as high as 70%. This highlights the critical need to safeguard the somatic nervous system during sepsis. While animal and human studies have demonstrated neuroinflammation, vascular changes, and metabolic disorders associated with SAE, a definitive pathophysiological mechanism remains elusive (4). Although SAE is classified as an acute complication of sepsis, studies reveal long-term cognitive impairment in survivors at rates exceeding 40%. Mazeraud et al. and Molnar et al. reported no complete recovery and persistent cognitive deficits in SAE patients, with the underlying cause for this long-term effect remaining unclear (4, 5). Interestingly, despite the infectious nature of sepsis, no direct infection is observed in the brains of these patients. Instead, the resulting neuroinflammation and oxidative stress are believed to be the primary contributors to the pathophysiology of SAE (6-9).

While initially developed to combat the influenza A virus, the emergence of resistance has limited the use of amantadine (AMA). However, its diverse pharmacological profile, including modulation of dopamine signaling and potential anticholinergic effects, has opened avenues for exploration for this NMDA receptor antagonist drug in various therapeutic areas. With its documented effectiveness in treating Parkinson’s disease, reducing neuronal damage from brain trauma or hemorrhage, and exhibiting potential anti-inflammatory properties, amantadine possesses a well-established safety profile with limited side effects and a convenient administration route. However, its use remains relatively uncommon (10-13). Given the limited research on SAE treatment in sepsis and based on AMA’s proposed mechanism of action, we hypothesized that it could be a potential therapeutic candidate (14,15). Therefore, this study aimed to evaluate the effectiveness of AMA in a rat model of LPS-induced sepsis.

## Materials and Methods

### Ethical approval

This study adhered to the ARRIVE (Animal Research: Reporting In Vivo Experiments) guidelines, v.2.0 protocol. The procedures performed on adult female Wistar albino rats (300-350 g) were reviewed and approved by the Suleyman Demirel University Animal Experiments Local Ethics Committee (ethical approval no. 11.05.2023-170/05). Additionally, Suleyman Demirel University Scientific Research Project Unit (SDU-BAP) provided financial support for the study (project no. TSG-2023-9092)

### Chemicals

The following chemicals were obtained from commercially available sources: LPS 100 (L2630 100 mg; Sigma-Aldrich, Burlington, MA, USA) and amantadine hydrochloride (A1260-5G; Sigma-Aldrich). In addition, XylazinBio 2% (Bioveta a.s, Ivanovice na Hane, Czech Republic) was used for sedation and Keta-Control (Doğa İlaç, Istanbul, Türkiye) for anesthesia.

### Animals

Thirty-two adult female Wistar albino rats (300–350 g) were housed in standard Euro type 4 cages under controlled conditions (23 °C, 55% humidity, 12 hr light/dark cycle) with *ad libitum* access to standard commercial feed and water. The animals were then divided into four experimental groups, with eight rats in each. 

**The control group r**eceived intraperitoneal (IP) injections of 0.5-1 ml of normal saline (NS) in the left inguinal region and 0.5 ml of NS in the right inguinal region 30 min apart.

**The LPS group re**ceived an IP injection of 0.5-1 ml NS in the left inguinal region, followed by an IP injection of 5 mg/kg LPS (16) in 0.5 ml NS in the right inguinal region 30 min later. Solid LPS was dissolved in NS.

**The LPS+AMA group r**eceived an IP injection of 45 mg/kg AMA (17) in 0.5-1 ml NS in the left inguinal region, followed by an IP injection of 5 mg/kg LPS in 0.5 ml NS in the right inguinal region 30 min later. 

Finally, the **AMA group r**eceived an IP injection of 45 mg/kg AMA in 0.5-1 ml NS in the left inguinal region, followed by an IP injection of 0.5 ml NS in the right inguinal region 30 min later.

Six hours after LPS administration, rats were euthanized by drawing blood from the vena cava inferior under anesthesia (90 mg/kg ketamine and 10 mg/kg xylazine) following abdominal incision. Brain tissues were collected and dissected. Right brain tissues were preserved in 10% formaldehyde solution for histopathological and immunohistochemical analyses. Left brain tissues were divided; one half was stored at -80 °C for genetic analysis, and the other half was stored at -20 °C for biochemical analysis.

### Biochemical analysis

Brain tissues were homogenized in a 1:5 dilution (w/v) with phosphate-buffered saline (10 mM sodium phosphate, pH 7.4) using a tissue homogenizer (IKA T25 ULTRA-TURRAX; IKA Werke GmbH & Co. KG, Staufen, Germany). Then, the homogenates were centrifuged at 2000 rpm for 20 min at +4 °C (Nüve NF 1200R; Nüve, Ankara, Turkey). Supernatants were used to assess tissue total anti-oxidant status (TAS) and total oxidant status (TOS) using an automated biochemistry analyzer (Beckman Coulter AU5800; Beckman Coulter, Inc., Brea, CA, USA) and colorimetric methods developed by Erel (18). TOS results were expressed as μmol H_2_O_2_ equivalent/g wet tissue, and TAS results were expressed as mmol Trolox equivalent/g wet tissue. The oxidative stress index (OSI) was calculated as TOS/TAS/10 (19).

### Histopathological evaluations

Brain, hippocampal, and cerebellar tissues were meticulously collected during necropsy and preserved in 10% buffered formalin for subsequent histological evaluation. The tissues were then processed by a fully automated tissue processor following established protocols. Subsequently, 5 μm-thick sections were obtained from paraffin blocks using a rotary microtome (Leica RM 2155; Leica Microsystems GmbH, Wetzlar, Germany). Before coverslipping, the sections underwent deparaffinization, ethanol rehydration, hematoxylin-eosin (HE) staining, and xylene washing. Under a light microscope, the histological changes were assessed in various brain regions using a semi-quantitative scoring system (0–3) to assess the extent of hyperemia, edema, hemorrhage, gliosis, and neuronal injury. Briefly, a score of 1 indicated lesions in less than 20% of the examined fields, 2 indicated lesions between 20% and 60%, and 3 indicated lesions in all examined fields.

### Immunohistochemical analysis

The current study employed immunohistochemistry to evaluate the expression of IFN-α (anti-interferon alpha 2 antibody, cat. no. ab193055), Caspase-3 (recombinant anti-Caspase-3 p12 antibody EPR16888, cat. no. ab179517), and TNF-α (TNF-α recombinant antibody RM1005, cat. no. ab307164) in brain tissues. Sections were mounted on poly-L-lysine slides (Abcam Ltd., Cambridge, UK) and processed using the streptavidin-biotin-peroxidase staining technique. Primary antibodies (all obtained from Abcam) were diluted 1:100. After sections were exposed to primary antibodies for an entire night, they were immunohistochemically stained using biotinylated secondary antibodies and streptavidin-alkaline phosphatase conjugate. A commercially available kit (EXPOSE Mouse and Rabbit Specific HRP/DAB IHC Detection kit, cat. no. ab80436; Abcam Ltd., Cambridge, UK) provided the secondary antibody and diaminobenzidine (DAB) as chromogen (Abcam Ltd., Cambridge, UK). The primary antiserum step utilized in the negative controls was swapped out for the antibody dilution solution. A qualified histopathologist from a different university carried out each analysis on blinded samples. At an objective magnification of ×40, the proportion of cells that were positively immunostained for each marker in ten different fields on each plate for each group was calculated. The ImageJ v.1.48 software (National Institutes of Health, Bethesda, MD, USA) was used to quantify the proportion of positively immunostained cells. Microphotographs were captured using the Database Manual Cell Sens Life Science Imaging Software System (Olympus Co., Tokyo, Japan).

### Reverse transcription-polymerase chain reaction (RT-qPCR)

Total RNA was isolated from homogenized brain tissues using the GeneAll® RiboEx™ RNA Isolation Kit (cat. no. 301-001; GeneAll Biotechnology Co., Ltd., Seoul, Korea) based on the manufacturer’s instructions. The BioSpec-nano spectrophotometer (Shimadzu Ltd., Kyoto, Japan) assessed RNA quantity and purity. cDNA synthesis was performed using the A.B.T.™ cDNA Synthesis Kit (cat. no. C03-01-05; Atlas Biotechnology, Ankara, Türkiye) according to the manufacturer’s protocol, with 1 μg of RNA per reaction. Specific mRNA sequences were identified using the Primer-BLAST tool on the NCBI website, and primer sequences were validated. Primer details (sequences, accession numbers, and product size) are provided in Table 1. A.B.T.™ SYBR Master Mix (catalog no. Q04-01-05; Atlas Biotechnology, Ankara, Türkiye) was used for gene expression quantification on a BioRad CFX96 Real-Time PCR Detection System (Bio-Rad Laboratories, Hercules, CA, USA). The Rn18s gene was used as a housekeeping gene in the analysis. The reaction mixture was prepared according to the manufacturer’s protocol for a final volume of 20 μl. The resulting reaction mixture was loaded onto a real-time qPCR instrument equipped with a thermal cycling setup, following the manufacturer’s protocol for the respective kit. Each sample underwent three independent replications for robust analysis. Relative mRNA levels were determined using the 2^−ΔΔCt^ formula to ensure data normalization. 

### Statistical analysis

Statistical analyses comparing groups were conducted via one-way ANOVA, followed by *post hoc* Tukey analysis for pairwise comparisons. GraphPad Prism software facilitated the statistical assessments, with significance set at *P*<0.05.

## Results

### Biochemical results

**Figure 1:**
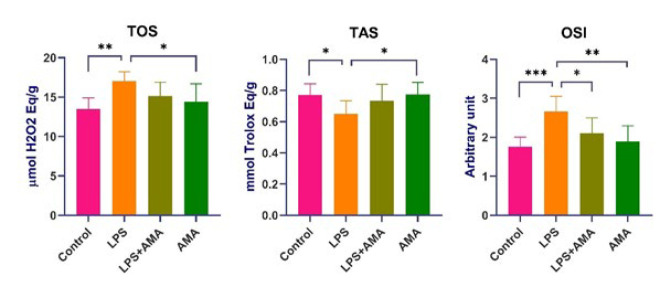
Oxidative stress parameters in brain tissues of rats

In the LPS group, TOS values exhibited a significant increase, while TAS values showed a significant decrease compared to the control group (*P*=0.045 and *P*=0.001, respectively). OSI values in the same group increased insignificantly compared to the control group (*P*<0.01). In the LPS+AMA group, OSI values decreased significantly compared to the LPS group. In the AMA group, TOS and OSI values decreased significantly compared to the LPS group (*P*=0.24 and *P*=0.01, respectively). Additionally, TAS values in the AMA group significantly increased compared to the LPS group (*P*=0.35) (Figure 1 ).

### Real-time qPCR results

**Figure 2:**
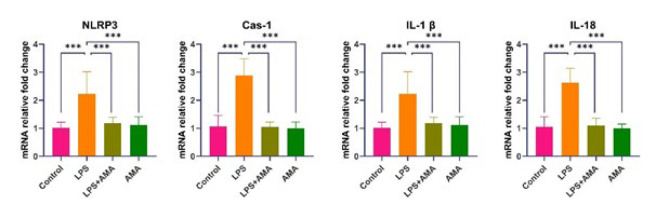
mRNA expression levels in brain tissues

In the LPS group, NLRP3, Cas-1, IL-1β, and IL-18 expressions showed a statistically significant increase compared to the control group. In contrast, a significant decrease was detected in the groups subjected to AMA treatment compared to the LPS group (*P*<0.001 for all) (Figure 2).

### Histopathological results

**Figure 3:**
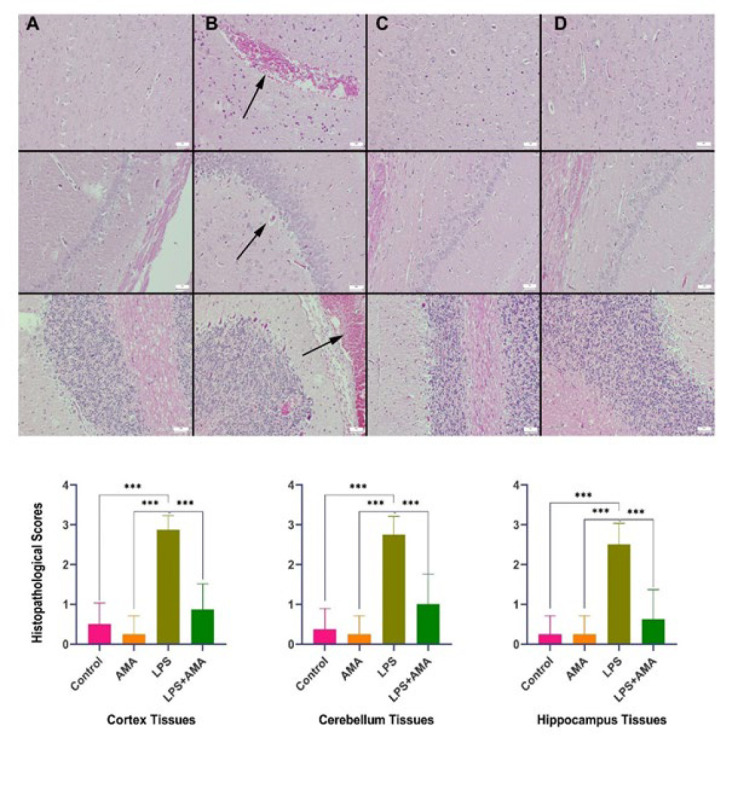
Histopathological variations observed in rats brain tissues (top row), rats cerebellum (middle row), and rats hippocampus (bottom row) across the groups

Histological assessment revealed normal findings in both the control and AMA groups. However, following LPS administration, the brain exhibited prominent signs of hyperemia, edema, hemorrhage, mild degeneration, neuronal death, and minimal gliosis. Additionally, inflammatory cell infiltrations were observed in the meninges of this group. Subsequent treatment with AMA ameliorated these lesions (Figure 3).

**Figure 4:**
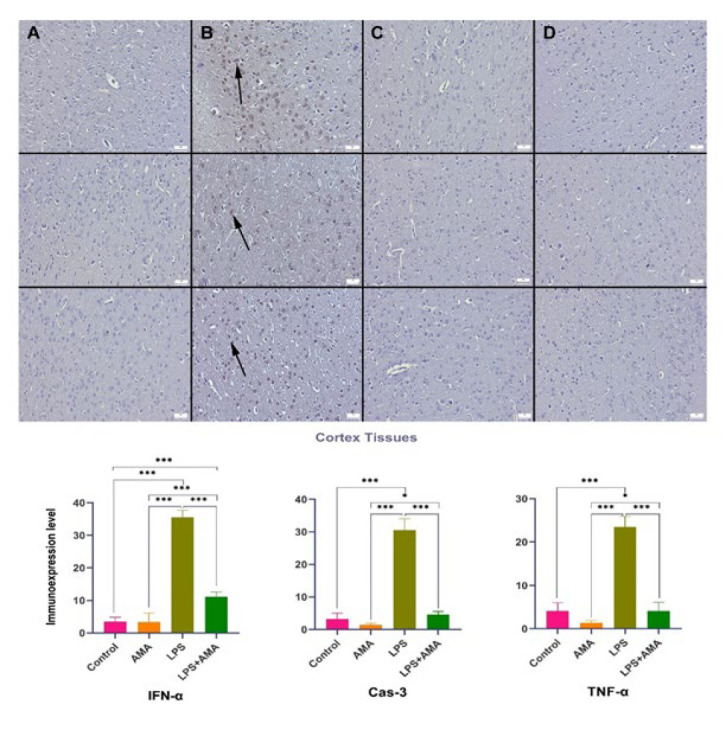
Expression levels of IFN-α (top row), Cas-3 (middle row), and TNF-α (bottom row) in the rats brain cortex tissue across the groups

**Figure 5:**
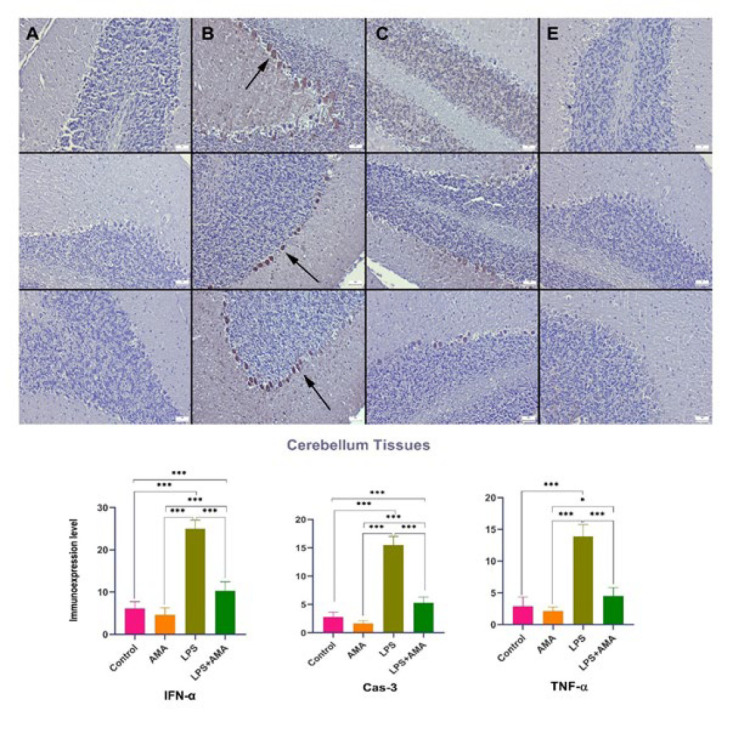
Expression levels of IFN-α (top row), Cas-3 (middle row), and TNF-α (bottom row) in rats cerebellum tissue among the groups

**Figure 6:**
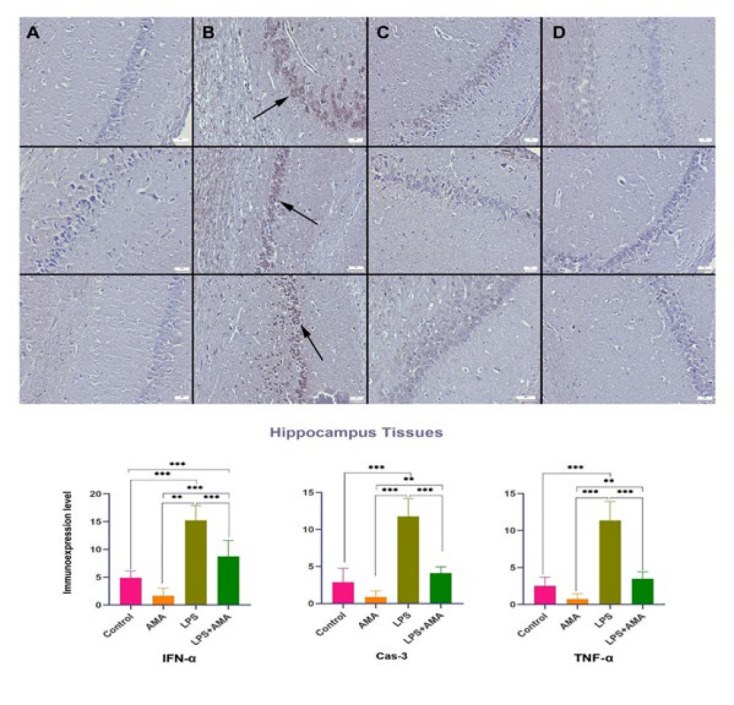
Expression levels of IFN-α (top row), Cas-3 (middle row), and TNF-α (bottom row) in rats hippocampus tissue across the groups

Upon examining the expressions of IFN-α, TNF-α, and Cas-3 in central nervous system neurons, it was noted that LPS injection led to a decrease in the conspicuous expression of IFN-α in the control group. Additionally, following LPS administration, there was a significant decrease in the expressions of TNF-α and Cas-3, which were either negligible or moderate in the control group. In the AMA group, IFN-α, Cas-3, and TNF-α expressions were nearly absent, contrasting with the strong expression observed in the brain, cerebellum, and hippocampal tissues of the LPS group. In the LPS+AMA group, AMA treatment normalized the expressions of IFN-α, Cas-3, and TNF-α (Figures 4-6).

The current study’s findings indicate that LPS induces abnormalities in the hippocampus, cerebellum, and brain regions, effectively mitigated by AMA treatment.

## Discussion

This study investigated the neuroprotective potential of AMA, a drug with antiviral and antiparkinsonian properties, against LPS-induced neuronal damage.

It is known that systemic inflammatory responses triggered by various factors, including bacterial or viral components, can damage multiple organs, including the brain. Prooxidant and proinflammatory mediators circulate in the bloodstream and bind to tissue receptors, leading to detrimental effects such as oxidative stress and inflammation (20). These mechanisms can culminate in organ dysfunction. Mortality often arises from complications related to affected organs. Increased permeability due to damage to the blood-brain barrier caused by circulating oxidants and inflammatory mediators can further compromise brain tissue (21). Such pathological processes in the brain contribute to neurological disorders like motor neuron damage, balance impairments, learning and memory deficits, and behavioral anomalies that impact morbidity (22, 23). Therefore, current research explores anti-oxidant and anti-inflammatory agents to mitigate these complications by targeting neuroinflammation, oxidative stress, mitochondrial dysfunction, and decreased brain metabolism (24, 25). 

In cases where the oxidant-anti-oxidant balance shifts in favor of oxidants, such as oxidative stress, indicators such as TOS and OSI increase in tissues (26). Our findings of increased TOS and OSI alongside decreased TAS levels in the LPS group suggest the development of oxidative stress in the injury group. Conversely, the reduction in OSI levels, particularly in the AMA treatment group, suggests a potential anti-oxidant effect of AMA. However, there was no significant increase in TAS in the group administered AMA alone compared to the control group, indicating that the drug may employ mechanisms beyond direct anti-oxidant activity. For instance, in the context of the mutually reinforcing relationship between oxidative stress and inflammation, AMA’s anti-inflammatory properties might indirectly contribute to the observed reduction in oxidative stress. Acknowledging that the effectiveness observed with the current acute model might differ with varying AMA doses or treatment durations is important.

The observed hyperemia, edema, hemorrhagic foci, and inflammatory cell infiltrates in the LPS group’s histopathological analysis corroborate the development of inflammation alongside the oxidative stress in brain tissue. Furthermore, the increased immunostaining of TNF-α, an indicator of acute inflammation, further supports this observation. The ability of AMA treatment to induce an acute regression in these markers suggests its potential efficacy in other disease models requiring acute interventions.

Acute infections often involve inflammation, which contributes to tissue damage. Bacterial-derived structures such as LPS stimulate intracellular pathways by activating proinflammatory signals in brain tissue via toll-like receptor 4 (TLR-4) (27). This signaling may lead to the expression of genes like nuclear factor kappa-beta (NF-κB), which is central to cytokine synthesis, initiating numerous inflammatory and apoptotic processes (28-30). In addition, pathways that trigger inflammation secondary to infection, such as the NLRP3 pathway, may also be activated. In this pathway, it is known that Cas-1, with increased expression observed in the LPS group, triggers inflammation by activating pro-IL-18 into mature IL-1β and IL-18 (31, 32). Studies on sepsis suggest that IL-1β is among the first inflammatory factors to increase and plays a crucial role in neurological dysfunction by mediating the entry of inflammatory mediators into the brain (33, 34). Consistent with these findings, our study demonstrated a significant decrease in IL-1β levels in the AMA group compared to the LPS group, suggesting that this reduction contributes to AMA’s neuroprotective activity.

The elevated TNF-α expression observed further supports the presence of inflammation arising from the NLRP3 pathway activation. The reduction in all these markers in the AMA treatment groups implies that AMA treatment can suppress the inflammatory response secondary to infection. Notably, AMA treatment also reduced the increased expression of IFN-α, a molecule with critical roles in acute inflammation and inflammasome activation, in LPS-treated rat brains. This finding suggests the drug’s potential efficacy in acute events and its ability to regress inflammasome activation. 

In some instances, such as infections with acute inflammation, tissue responses may indicate progressive damage that could culminate in a more severe chronic process. In this study, AMA treatment reversed the histopathologically detected degenerative changes, neuronal cell death, and increased Cas-3 levels (a marker of programmed cell death or apoptosis) observed in the LPS group. This reversal suggests that AMA could potentially prevent neurodegenerative diseases that might develop chronically. Additionally, it might offer protection against neurological dysfunction arising from acute apoptotic processes. Similar to our findings, Xing *et al*. (35) demonstrated that AMA treatment alleviated learning and memory deficits in sepsis rats and reduced neuroinflammation. This supports the notion that AMA exerts its neuroprotective effects through various mechanisms.

**Table 1:** Primary sequences, product size, and accession numbers of genes.examined in rat tissues

**Genes**	**Primary sequence**	**Product size**	**Accession number**
**Rn18s (housekeeping gene)**	F: CTCTAGATAACCTCGGGCCG	209 bp	NR_046237.2
	R: GTCGGGAGTGGGTAATTTGC		
**IL-1β**	F: TTGAGTCTGCACAGTTCCCC	161 bp	NM_031512.2
	R: GTCCTGGGGAAGGCATTAGG		
**Caspase 1**	F: GACCGAGTGGTTCCCTCAAG	108 bp	NM_012762.3
	R: GACGTGTACGAGTGGGTGTT		
**NLRP3**	F: TCTCTGCATGCCGTATCTGG	295 bp	NM_001191642.1
	R: ACGGCGTTAGCAGAAATCCA		
**IL-18**	F: TCACTTCAGTGTCTCTGTGAGC	95 bp	NM_019165.2
	R: TTCCAACTGAGAGGCTGTGC		

Rn18s: 18S ribosomal RNA, IL-1β: interleukin-1 beta, Caspase 1: cysteine-aspartic proteases 1, NLRP3: NLR family pyrin domain containing 3, IL-18: interleukin-18, F: forward, R: reverse.

TOS: Total oxidant status, TAS: Total anti-oxidant status, OSI: Oxidative stress index, LPS: Lipopolysaccharide, AMA: Amantadine. *P<0.05, **P<0.01, ***P<0.001

NLRP3: NLR family pyrin domain containing 3, Cas-1: Cysteine-aspartic proteases 1, IL-1β: Interleukin-1 beta, IL-18: Interleukin-18.*P<0.05, **P<0.01, ***P<0.001

(A) Normal tissue histology in the control group. (B) Marked hemorrhage and hyperemia (arrows) in the LPS group. (C) Reduced histopathological lesions in the LPS+AMA group. (D) Normal tissue histology in the AMA group. H&E staining, scale bars = 50 µm.

(A) Negative to slight IFN-α, Cas-3, and TNF-α expressions in the control group. (B) Increased IFN-α, Cas-3, and TNF-α expressions (arrows) in the LPS group. (C) Decreased IFN-α, Cas-3, and TNF-α expressions in the LPS+AMA group. (D) Negative to slight IFN-α, Cas-3, and TNF-α expressions in the AMA group. Streptavidin-biotin-peroxidase staining, scale bars = 50 µm.

(A) No IFN-α, Cas-3, and TNF-α expression in the control group. (B) Increased IFN-α, Cas-3, and TNF-α expressions (arrows) in the LPS group. (C) Decreased IFN-α, Cas-3, and TNF-α expressions in the LPS+AMA group. (D) Marked IFN-α, negative to slight Cas-3 and TNF-α expressions in the AMA group. Streptavidin-biotin-peroxidase staining, scale bars = 50 µm.

(A) Negative to slight IFN-α, Cas-3, and TNF-α expressions in the control group. (B) Increased IFN-α, Cas-3, and TNF-α expressions (arrows) in the LPS group. (C) Decreased IFN-α, Cas-3, and TNF-α expressions in the LPS+AMA group. (D) Negative to slight IFN-α, Cas-3, and TNF-α expressions in the AMA group. Streptavidin-biotin-peroxidase staining, scale bars = 50 µm.

## Conclussion

The current study provides evidence for the potential efficacy of AMA in mitigating sepsis-related brain damage. Future studies confirming these effects of AMA treatment across various disease models exhibiting NLRP3 pathway suppression would support its application in broader clinical settings. Given its established safety and efficacy profile, AMA emerges as a promising therapeutic candidate for SAE. However, further clinical and experimental investigations are warranted to establish its role in this context definitively.

## Declaration

The datasets generated and/or analyzed in this study are available from the corresponding author upon reasonable request. We have not used any AI tools or technologies to prepare this manuscript.
